# Fluorine-Modulated Electronic Structure and Atomic Bonding of the Titanium Surface

**DOI:** 10.3390/ma15238492

**Published:** 2022-11-29

**Authors:** Lei Li, Haihua Huang

**Affiliations:** 1 Key Laboratory of Extraordinary Bond Engineering and Advanced Materials Technology (EBEAM) of Chongqing, Yangtze Normal University, Chongqing 408100, China; 2 School of Materials Science and Engineering, Liaocheng University, Liaocheng 252059, China

**Keywords:** fluorine, Ti(0001), DFT, adsorption, entrapment, polarization

## Abstract

The fluorine-adsorption-induced local bond relaxation and valence-energy-state evolution of the Ti(0001) surface were examined through density functional theory calculations. The predicted bond–band–barrier (3 B) correlation notation framework for the interaction of the fluorine adsorbate with Ti atoms formed a tetrahedral structure through the creation of four valence density-of-state features, namely bonding electron pairs, nonbonding lone pairs, holes, and antibonding dipoles. The bonding states resulted in the passivation of metal Ti surfaces, the formation of Ti^p^ dipoles and Ti^+/p^ H-like bonds modulated the work function of the Ti(0001) surface, and the conversion of metallic Ti to semiconducting titanium fluoride by the holes. The findings of this study confirm the universal applicability of the 3 B correlation notation in the dynamics of fluorine chemisorption and the associated valence electrons involved in fluorination.

## 1. Introduction

The field of fluorination chemistry has existed for over 100 years and is now amenable to a wide range of applications, such as pharmaceuticals [1,2], agrochemicals [3,4], and materials [5,6,7,8,9]. Most fluorination reactions still lack generality, predictability, and cost-efficiency. An atom of fluorine, which is the most electronegative element, readily acquires electrons and undergoes *sp* orbital hybridization upon interactions with metals and thus exhibits diverse macroscopic properties. Although metal fluorination has been intensively investigated and widely applied, the relationships between local atomic bonding, electronic structures, and the observed or predicted properties of the resulting products and their interdependence are still unclear. Therefore, the relationship between chemical bonds and the valence density of states (DOSs) and their effects on material properties require further investigation.

Upon interacting with metallic Ti, O, and N receive two and three electrons from Ti, respectively, and *sp* orbital hybridization occurs. The difference in the number of bonding and lone pair electrons determines the sites at which O and N atoms are adsorbed on metal surfaces and the structures of the resulting compounds. The adsorption of O and N on the Ti(0001) surface has been investigated both experimentally and computationally [10,11,12,13,14,15,16,17,18,19]. The O atoms preferentially occupy the surface fcc sites. With increasing coverage of the surface by O, non-adjacent layers of the Ti(0001) surface are gradually occupied owing to mutual repulsion between additional lone pairs of electrons associated with the adsorbed O atoms [18]. Conversely, N atoms first adsorb at oct(1,2) sites to form a stable (1 × 1) structure. Once these sites are fully occupied, an additional (√3 × √3) structure forms on the hcp sites on the top surface with increasing N coverage [12,19]. The work function initially decreases and then increases with increasing adsorbate coverage owing to the formation of hydrogen-bond-like interactions. Similarly, electrons should readily undergo *sp* orbital hybridization upon interaction with the metal owing to the electronic structure of the fluorine atom. Furthermore, fluorine atoms are more electronegative than O and N atoms. The *sp*-hybridized fluorine atom that interacts with the metal has one pair of bonding electrons and three nonbonding lone pairs of electrons, which affect the adsorption position on the surface and change the electronic structure and work function of the metal surface.

Herein, we conducted density functional theory (DFT) calculations to analyze four DOS features of the Ti(0001) surface representing bonding electron pairs, nonbonding lone pairs, holes, and metal antibonding dipoles. The adsorption of fluorine was found to polarize the conduction electrons through its nonbonding states and lower the work function of the Ti(0001) surface, while the generation of holes transformed the conductor into a semiconductor.

## 2. DFT Methods

DFT calculations were performed using the CASTEP code [20] with spin polarization. Ultrasoft pseudo-potentials were employed to describe the core electrons, which allowed for a lower energy cutoff than the norm-conserving potentials. The exchange and correlation interactions were subjected to generalized gradient approximation (GGA) with Perdew–Wang parameterization (known as GGA-PW91) [21]. In all calculations, an energy cutoff of 440 eV was employed to limit the expansion of plane-waves while the Brillouin zone was integrated using special k-points generated with a 15 × 15 × 15 mesh parameter grid for Ti primitive cells and a 6 × 6 × 1 grid for the surface and interlayers of Ti(0001) with a (2 × 2) supercell. The lattice constant of the bulk Ti and the interlayer relaxation of the clean Ti(0001) surface were determined to estimate calculational accuracy. The above simulation determined that the lattice parameters a and c/a of the bulk Ti were 2.935 Å and 1.581, respectively; these values should be compared with the experimental values of 2.95 Å and 1.586, as reported in Ref. [22], and with the values of 2.94 Å, 1.591 obtained using GGA [23].

The Ti(0001) surface was obtained by cutting a slab of three times the lattice thickness in the <0001> direction from an optimized Ti single cell and applying a vacuum layer of 15 Å. The lowermost two Ti atoms were fixed during structural optimization, and the dipole moment was corrected to account for the slab asymmetry [24].

The spacing of the outermost layer contracts by approximately 6.03% when the (0001) slab is relaxed while those of the second and third layers contract by approximately 2.59% and 0.43%, respectively. These results are consistent with experimentally obtained data [25] and the expected bond order–length–strength (BOLS) correlation mechanism [26], which stipulates that bonds between the undercoordinated atoms become shorter and stronger.

## 3. Results

### 3.1. Adsorption Sites and Work Functions

**Figure 1:**
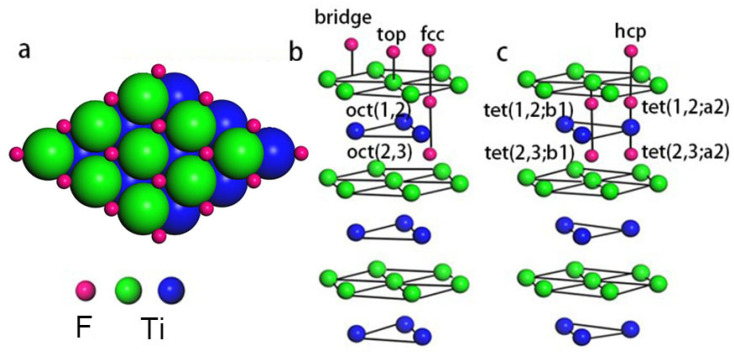
(**a**) Top view of the Ti(0001) surface with F atoms occupying all fcc sites. (**b**,**c**) Perspective views of the F adsorption sites; oct(*i,j*) denotes an octahedral site between the *i*th and (*i* + 1)th layers, and tet(*i*,:a*j*(or b*i*)) denotes a tetrahedral site located directly below the *i*th layer.

All possible adsorption positions on the Ti(0001) surface are shown in Figure 1b,c. Based on previous calculations, we first examined all possible adsorption positions to determine the most stable adsorption position for a single F atom based on the magnitude of the adsorption energy at an adsorption coverage of 0.25 ML. The amount of adsorbed fluorine was gradually increased based on this result, and the adsorption energy was calculated at different positions after structural optimization. The most likely stable F adsorption site is the location with the highest adsorption energy according to the following equation:

$$E_{ads}=\frac{1}{N}\left(E_{clean}+N\mu_{F}-E_{F/Ti\left(0001\right)}\right),\;\mu_{F}=E_{HF}-\frac{1}{2}E_{H_{2}},$$

where *N* is the number of adsorbed F atoms, and *E_F/Ti(0001)_*, *E_clean_*, *E_HF_,* and *E_H2_* are the total energies of the F-adsorbed Ti slab, clean Ti slab, single HF molecule, and single H_2_ molecule, respectively.

The work function of the clean surface was 4.47 eV, consistent with experimentally determined values (4.45–4.60 eV) [27] and previous DFT results (4.45 ± 0.01 eV) [15,28].

Repeated optimizations yielded average adsorption energies for the fluorine atoms and work functions, average F-Ti bond lengths, and charge Mulliken populations for the most stable adsorption position (Table 1).

The surface fcc site is the most stable location for the adsorption of atomic fluorine on the Ti(0001) surface during the initial adsorption stage (i.e., at 0.25 ML coverage). The hcp site is slightly higher in energy than the fcc site, whereas the top, bridge, and tetrahedral sites are energetically unfavorable. F atoms preferentially occupy the fcc sites on the surface and do not enter the interior of the Ti atomic layer until all of the surface fcc sites are filled with increasing F-atom coverage to form a stable (1 × 1) structure at a saturation coverage of 1.00 ML (Figure 1a). Unlike the multilayer adsorption of O and N, which occurs at high coverages, fluorine atoms adsorb only at surface fcc sites because lower Ti surface layers are too energetic to allow for F-atom adsorption.

**Figure 2:**
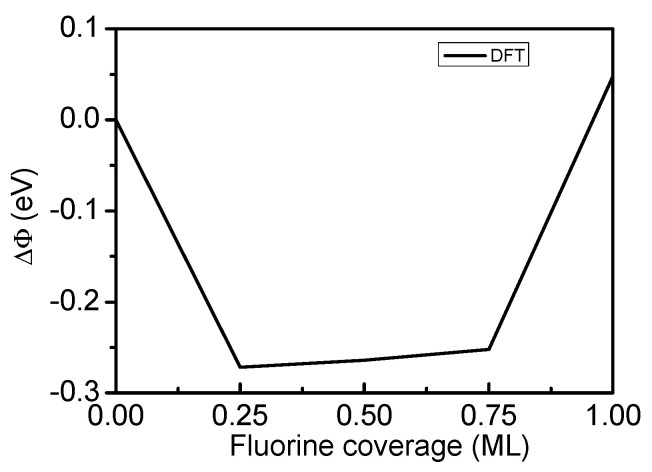
Change in the work function of the F-Ti(0001) surface as a function of fluorine coverage.

The changes in the Ti(0001)-surface work function (Δϕ) are shown in Figure 2 and Table 1, along with the calculated work function of the clean Ti(0001) surface (4.47 eV) as a reference. The work function exhibits a significant reduction owing to the adsorption of F atoms on the Ti(0001), remains essentially constant between 0.25 and 0.75 ML, and then significantly increases with increasing F-atom adsorption up to 1.00 ML.

The average bond length between F and the three adjacent Ti atoms decreased between 0.25 and 0.75 ML but significantly increased with increasing adsorption coverage to 1.00 ML. This is consistent with the observed trend for the work function. In contrast, the charge acquired by the F from the Ti remained constant at 0.46, according to Mulliken population analysis.

### 3.2. Electronic Structure

**Figure 3:**
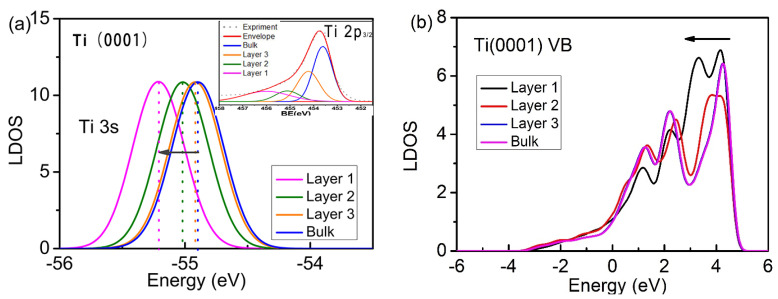
Layer-resolved LDOS of Ti3s (**a**) and valence band (**b**) of the Ti(0001) clean surface. Arrows indicate a shift in binding energy to deeper energy levels as the coordination number decreases, i.e., quantum entrapment. (a, inset): experimentally determined layer-resolved XPS resolution spectrum of the Ti2p core level. The energy shift of each component is proportional to the magnitude of bond energy according to the following relation: $\Delta E_{v}\left(i\right)/\Delta E_{v}\left(12\right)=E_{i}/E_{B}=C_{i}^{-m}\left(i=S_{1},S_{2},\ldots ,B\right)$ [29].

An electronic structure analysis of the non-adsorbed clean Ti(0001) surface was conducted, along with an analysis of the local DOSs (LDOSs) of the Ti3s energy levels and the valence electrons in different layers of the surface (Figure 3). Layer 1, layer 2, layer 3, and B represent the electron DOSs of the Ti atoms in the first, second, and third layers of the surface and in the bulk, respectively. The atomic coordination number tends to decrease upon moving from the bulk to the surface. In agreement with the X-ray photoelectron spectroscopy (XPS) 2p_3/2_ core energy level spectra (Figure 3, inset) obtained by deconvolution experiments, the DOS of either the valence or Ti3s deep energy level core electrons undergoes quantum entrapment as the coordination number decreases toward the deep energy level. This is in full agreement with the projections of the BOLS theory.

**Figure 4:**
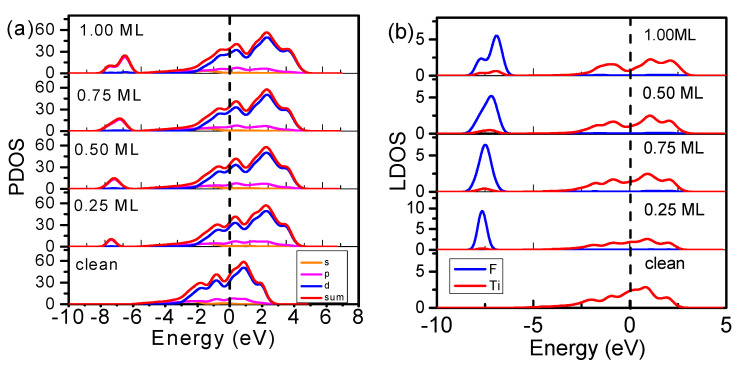
Ti(0001)-F coverage dependence of (**a**) valence projected DOSs and (**b**) valence local DOSs (LDOSs) of the most stable adsorption sites. The blue and red lines in represent the average LDOS per F and Ti atom in the surface area, respectively. E = 0 eV is the reference Fermi energy.

**Figure 5:**
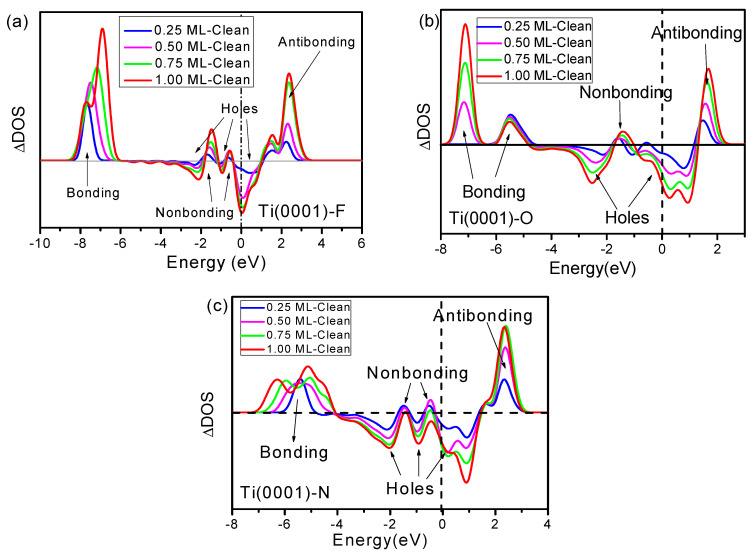
(**a**) Changes in the DOS (ΔDOSs) of the Ti(0001) surface at various F-atom adsorption concentrations compared to (**b**) O atom adsorption and (**c**) N atom adsorption. Three adsorbed atoms led to the same four-electron structure change features corresponding to antibonding dipoles, nonbonding states, bonding states, and holes.

To analyze the changes in the electronic structure of the Ti(0001) surface resulting from the concentration of F atoms adsorbed at the most stable adsorption sites (fcc sites), the valence projected DOSs and LDOSs of the Ti atoms interacting directly with the F atoms were analyzed at different concentrations (0, 0.25, 0.50, 0.75, and 1.00 ML), as shown in Figure 4. For visual comparison, the LDOS of both Ti and F in Figure 4b are averaged per atom. Fluorine exchanges and polarizes the electrons of the Ti atoms rather than simply adding its *2s* and *2p* electrons to the valence band of the Ti metal. To observe changes in the electronic structure of the Ti surface due to F-atom adsorption more clearly, we examined the differences in the DOS before and after adsorption (Figure 5a). Fluorine adsorption led to four obvious DOS features between +1.0 and +4.0 eV (antibonding), at approximately −1.0 eV (nonbonding), between −3.0 and +1.0 eV (holes), and between −6.0 and −8.0 eV (bonding), which are surprisingly consistent with the DOS measurements of the O-Ti(0001) and N-Ti(0001) skins (Figure 5b,c).

The DOS features between −8.0 and −6.0 eV arise from derivative Ti-F bonds that are stronger than the original Ti-Ti bonds, which stabilizes the system by lowering its overall energy. Fluorine passivates metal surfaces, while fluorides exhibit excellent chemical and thermal stabilities and reliable mechanical performance at high temperatures. The lone pair of fluorine moves the d-electron of Ti toward low energy, thereby giving rise to the antibonding states between +1.0 and +4.0. Furthermore, the locally densified and entrapped bonding and core electrons in the undercoordinated surface atoms further increase dipole polarity. The formation of antibonding states results in a reduction in the work function of the Ti surface (e.g., from 0.25 to 0.75 ML F-atom adsorption concentration). As the concentration of F atoms increases further, a portion of the polarized Ti participates in bonding with F, forming a hydrogen-bond-like interaction and thereby increasing the work function. This explains the significant increase in the work function of the system at 1.00 ML concentration adsorption and the appearance of two peaks of different energy magnitudes in the bonding state. Movements of the deep bonding state and shallow antibonding state promote the formation of holes near the Fermi level, which transforms metallic Ti(0001) into semiconducting Ti_x_F_y_, such as TiF_4_, TiF_2_, and TiF_6_. This mechanism provides further theoretical support for future research into the modulation of bandgaps by fluorides. The DOS features near the Fermi energy level are attributable to the effects of the non-bonded lone pair electrons and holes.

## 4. Discussion

The F-atom adsorption-induced changes in local bonding and the electronic structure correlations on the metal surfaces are in full agreement with the predictions of 3 B correlation theory [26]: when C, N, O, and F interact with elements with lower electronegativity, they receive a corresponding number of electrons to fill their *2p* shell layers and thus undergo *sp* orbital hybridization. The numbers of bonding electron pairs and nonbonding electron lone pairs determine the tetrahedral coordination configurations with different symmetries in the local area of the adsorbed atom. This local bonding structure determines four additional electronic structural features, including bonding, nonbonding lone pairs, holes, and antibonding dipoles, which control the chemical and physical properties of the chemisorbed skin.

The fluorine atom, for example, captures one electron from an arbitrary Z-element that is less electronegative than F as they interact in the solid phase. Eight electrons occupying the full *2s* and *2p* levels of F undergo *sp* orbital hybridization, and these hybridized electrons form four oriented orbitals. One of these hybridized orbitals contains shared electron pairs, while the other three contain lone electron pairs, leading to the quasi-tetrahedral C_3v_ symmetry. Similarly, C interacts with Z to trap four electrons, while N and O trap three and two electrons, respectively. C, O, and N all form tetrahedral coordination structures.

**Figure 6:**
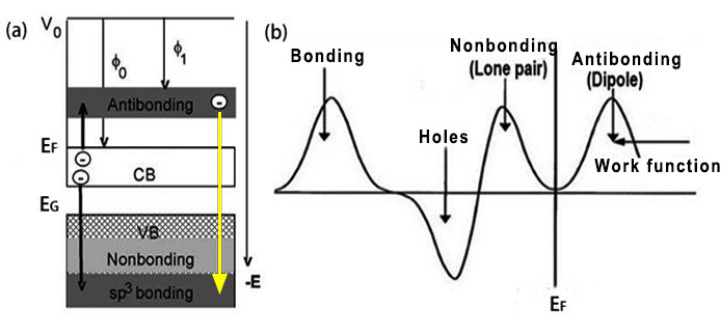
Schematic illustration showing the electronic dynamics associated with fluoridation (**a**) and the four valence DOS features (**b**). The arrows in (**a**) show that F captures electrons from one Z neighbor to form nonbonding lone pairs. These lone pairs induce dipole formation by polarizing the valence electrons of another three neighbors. Further F dosing captures the dipole to continue the bonding processes. The yellow arrow from the antibonding states to the bonding states highlights the process of the formation of H-bond-like interactions [26].

Fluoridation creates four additional electronic features in the valence electron of the host in the following manner (Figure 6a):

The electron is transferred from Z to F, occupying the full 2s^2^2p^4^ orbital of the F atom, and the energy of these electrons is slightly below the 2p energy level of F before orbital hybridization occurs.

The formation of F-Z bonds results in *sp* hybridization, which creates nonbonding lone pair states at energies slightly lower than the Fermi level (*E_F_*).

The lone pair of electrons will polarize the unpaired electrons in the adjacent Z atom, causing them to move to a shallower energy level and thus occupy the antibonding orbital.

The F-Z bond moves electrons toward deep energy levels while the antibonding state moves to the shallow energy level, resulting in the formation of holes near the Fermi level.

The formation of bonding states reduces the energy of the system and passivates the host system; the formation of antibonding states reduces the distance to the vacuum energy level and thus reduces the work function of the host system; the formation of holes gradually opens the band gap and thus changes the conductivity of the system; and the nonbonding lone pairs do not follow the conventional dispersion relationship but introduce impurity states near the *E_F_*, which together with the holes, determines the electronic structure near the *E_F_*. When overdosed with fluorine, a portion of the polarized dipole also participates in bonding; as indicated by the yellow arrow in Figure 6a, Z^p^ is converted to Z^+/p^ (i.e., H-bond-like formation), which leads to a re-increase in the previously reduced work function.

## 5. Conclusions

We estimated the atomic bonding and electronic structure of F atoms adsorbed on Ti(0001) surfaces using 3B theory and DFT calculations. The following results were obtained:

(1) In agreement with the predictions of BOLS theory and experimental XPS resolution spectra, the presence of undercoordinated atoms at the Ti(0001) surface reduces the local bond length and deepens the electronic energy. F adsorbed on the Ti(0001) surface only occupies the surface fcc sites.
(2) Consistent with expectations based on 3B theory, DFT calculations demonstrated the necessity of the tetrahedral F–Ti structure formed through the adsorption of fluorine on the Ti(0001) surface, with four additional DOS features. These features correspond to F−Ti bonding, F electron lone pairs, Ti^+^ electron holes, and Ti antibonding dipoles, which determine the physical and chemical properties of the chemisorbed surface. The F–Ti bonding states lower the energy of the system and passivate the metal surface while the creation of holes can transform metallic Ti into a semiconductor upon the adsorption of fluoride. The antibonding dipoles and H-bond-like interactions modify the work function in a manner dependent on the fluorine adsorption coverage and the adsorption sites. These findings are consistent with the measurements of the adsorption of O and N on Ti(0001) skin.

These observations provide a general understanding of the fluoridation process and will inform the design and fabrication of future fluoride-based functional materials.

## Figures and Tables

**Table 1:** Average adsorption energies per F atom at the various adsorption sites of the Ti(0001) surface, along with the work function, average Ti-F bond length, and transferred charge. The work function of the clean Ti(0001) surface is 4.47 eV.

Adsorption Coverage	Adsorption Site	Adsorption Energy (eV)	Work Function (eV)	$\mathrm{Ti}-F\;\mathrm{Bond}\;\mathrm{Length}\;(d_{F})$	Charge Transfer (e) (F → Ti)
0.25	fcc	2.93	4.198	2.098	0.46
	hcp	2.72	4.19		
	bridge	-	-		
	top	1.92	5.21		
	oct (1,2)	0.35	3.84		
	tet(1,2; b1)	−0.29	4.385		
	tet(1,2; a2)	-	-		
	oct(2,3)	1.09	4.371		
0.50	fcc	2.91	4.206	2.112	0.46
	oct(1,2)	0.54	4.20		
	fcc + oct(1,2)	1.64	4.22		
	hcp + oct(1,2)	1.61	4.21		
	fcc + tet(1,2; b1)	-	-		
	fcc + oct(2,3)	2.04	4.215		
0.75	fcc	2.86	4.218	2.131	0.46
	oct(1,2)	0.52	4.197		
	fcc + oct(1,2)	1.99	4.207		
1.00	fcc	2.81	4.518	2.109	0.46
	fcc + oct(1,2)	1.60			
	fcc + oct(2,3)	1.91			

## References

[1] Muller K., Faeh C., Diederich F. (2007). Fluorine in pharmaceuticals: Looking beyond intuition. Science.

[2] Böhm H.J., Banner D., Bendels S., Kansy M., Kuhn B., Müller K., Obst-Sander U., Stahl M. (2004). Fluorine in medicinal chemistry. ChemBioChem.

[3] Jeschke P. (2004). The unique role of fluorine in the design of active ingredients for modern crop protection. ChemBioChem.

[4] Maienfisch P., Hall R.G. (2004). The importance of fluorine in the life science industry. CHIMIA Int. J. Chem..

[5] Magnussen O.M. (2002). Ordered Anion Adlayers on Metal Electrode Surfaces. Chem. Rev..

[6] Marković N.M., Ross P.N. (2002). Surface science studies of model fuel cell electrocatalysts. Surf. Sci. Rep..

[7] Babudri F., Farinola G.M., Naso F., Ragni R. (2007). Fluorinated organic materials for electronic and optoelectronic applications: The role of the fluorine atom. Chem. Commun..

[8] Cametti M., Crousse B., Metrangolo P., Milani R., Resnati G. (2012). The fluorous effect in biomolecular applications. Chem. Soc. Rev..

[9] Berger R., Resnati G., Metrangolo P., Weber E., Hulliger J. (2011). Organic fluorine compounds: A great opportunity for enhanced materials properties. Chem. Soc. Rev..

[10] Eastman D.E. (1972). Photoemission energy level measurements of sorbed gases on titanium. Solid State Commun..

[11] Shih H.D., Jona F. (1976). Atomic underlayer formation during the reaction of Ti {0001} with nitrogen. Surf. Sci..

[12] Shih H.D., Jona F., Jepsen D.W., Marcus P.M. (1976). Low-Energy-Electron-Diffraction Determination of the Atomic Arrangement in a Monatomic Underlayer of Nitrogen on Ti (0001). Phys. Rev. Lett..

[13] Brearley W., Surplice N.A. (1977). Changes in the work function of titanium films owing tothe chemisorption of N_2_, O_2_, CO and CO_2_. Surf. Sci..

[14] Fukuda Y., Elam W.T., Park R.L. (1978). Nitrogen, Oxygen, and Carbon Monoxide Chemisorption on Polycrystalline Titanium Surfaces. Appl. Surf. Sci..

[15] Feibelman P., Himpsel F. (1980). Spectroscopy of a surface of known geometry: Ti (0001)-N(1×1). Phys. Rev. B.

[16] Biwer B.M., Bernasek S.L. (1986). A photoelectron and energy-loss spectroscopy study of Ti and its interaction with H_2_, O_2_, N_2_ and NH_3_. Surf. Sci..

[17] Kuznetsov M.V., Shalaeva E.V. (2004). Competing Adsorption of Nitrogen and Oxygen at the Ti (0001) Face: XPS Examination. Phys. Met. Metallogr..

[18] Li L., Meng F., Tian H., Hu X., Zheng W., Sun C.Q. (2015). Oxygenation mediating the valence density-of-states and work function of Ti (0001) skin. Phys. Chem. Chem. Phys..

[19] Li L., Meng F.L., Hu X.Y., Qiao L., Sun C.Q., Tian H.W., Zheng W.T. (2016). Nitrogen mediated electronic structure of the Ti (0001) surface. RSC Adv..

[20] Hoffmann M.R., Martin S.T., Choi W., Bahnemann D.W. (1995). Environmental applications of semiconductor photocatalysis. Chem. Rev..

[21] Perdew J., Ziesche P., Eschrig H., Ziesche P., Eschrig H. (1991). Chapter Unified Theory of Exchange and Correlation beyond the Local Density Approximation. Electronic Structure of Solids.

[22] Kittel C. (1996). Introduction to Solid State Physics.

[23] Schneider J., Ciacchi L.C. (2010). First principles and classical modeling of the oxidized titanium (0001) surface. Surf. Sci..

[24] Bengtsson L. (1991). Dipole correction for surface supercell calculations. Phys. Rev. B.

[25] Shih H.D., Jona F., Jepsen D.W., Marcus P.M. (1976). The structure of the clean Ti (0001) surface. J. Phys. C Solid State Phys..

[26] Sun C.Q. (2014). Relaxation of the Chemical Bond.

[27] Hanson D., Stockbauer R., Madey T. (1981). Photon-stimulated desorption and other spectroscopic studies of the interaction of oxygen with a titanium (001) surface. Phys. Rev. B.

[28] Huda M., Kleinman L. (2005). Density functional calculations of the influence of hydrogen adsorption on the surface relaxation of Ti (0001). Phys. Rev. B.

[29] Li L., Tian H.W., Meng F.L., Hu X.Y., Zheng W.T., Sun C.Q. (2014). Defects improved photocatalytic ability of TiO_2_. Appl. Surf. Sci..

